# Transcriptome analysis for the identification of cellular markers related to trabecular meshwork differentiation

**DOI:** 10.1186/s12864-017-3758-7

**Published:** 2017-05-17

**Authors:** Padmapriya Sathiyanathan, Cheryl Y. Tay, Lawrence W. Stanton

**Affiliations:** 1 Genome Institute of Singapore, Agency for Science, Technology and Research (A*STAR), Singapore, Singapore; 2 School of Biological Sciences, Nanyang Technological University, Singapore, Singapore; 3 Department of Biological Sciences, National University of Singapore, Singapore, Singapore

**Keywords:** Human trabecular meshwork, Markers, Differentiation, Glaucoma

## Abstract

**Background:**

Development of primary open-angle glaucoma (POAG) is associated with the malfunctioning trabecular meshwork (TM). Cell therapy offers great potential for the treatment of POAG, but requires the generation of functional TM cells in vitro to replace the lost/dysfunctional cells. TM differentiation in vitro from various stem cell types must be monitored by the expression of specific markers. However, no single definitive marker of the TM has been identified.

**Results:**

To identify robust markers of TM differentiation, we performed global transcriptome profiling using high-density oligonucleotide microarray on *ex vivo* TM tissue and cultured TM progenitors. Corneal and scleral tissues were also used in the analysis. After removal of genes expressed in the cornea and sclera, 18 genes were identified that were differentially expressed in the TM relative to the other samples. *CDH23*, F5, *KCNAB1*, *FGF9*, *SPP1*, and *HEY1* were selected among the genes highly expressed in the TM, together with *BDNF* which was repressed, compared to progenitors for further investigation. Expression analysis by qPCR verified the differential expression and immunofluorescence of the anterior segment confirmed strong expression in the TM.

**Conclusions:**

Three independent cohort of expression studies have identified novel markers, fitting in identifying TM cells and in evaluating directed TM differentiation in vitro.

**Electronic supplementary material:**

The online version of this article (doi:10.1186/s12864-017-3758-7) contains supplementary material, which is available to authorized users.

## Background

Primary open-angle glaucoma (POAG) is a leading cause of irreversible blindness worldwide. It is defined by a characteristic pattern of damage to the optic nerve head resulting in a progressive loss of vision. Elevated intraocular pressure (IOP) is an important risk factor for axonal damage at the optic nerve head [1–3]. In POAG patients, abnormalities in the trabecular meshwork (TM) lead to increased resistance to aqueous humour drainage and impedes outflow from the anterior chamber [4–6].

The TM located at the corneoscleral limbus is responsible for draining approximately 90% of the aqueous humour from the anterior chamber via conventional outflow pathway [7]. Alterations in the composition and deposition of extracellular matrix (ECM) together with significant decline in the number of TM cells have been observed in the diseased tissue of POAG specimens [8–13]. Accumulation of the ECM components, fibronectin, elastin and fibrillar material change the ECM composition [8, 9]. Glycosaminoglycan profiling also found an increase in chondroitin sulfate and decline in hyaluronan in the diseased tissues [10, 11]. Other structural modifications include thickening and fusion of the trabeculae, and deposition of sheath-derived (SD) plaques [8, 12]. Furthermore, remnant cells in the meshwork seem to be under stress, with increased expression of stress proteins, crystallin alpha B and inducible nitric oxide synthase [13]. Genome-wide expression studies found altered expression of certain genes with the pathology as well, such as those pertaining to inflammation, acute-phase response and G-protein signalling being elevated, while antioxidants and members of the solute carrier family were downregulated among others [14]. Such aberrations in the TM are believed to interfere with its filtering mechanism and its ability to control IOP.

Recent work has established the therapeutic potential of TM cell replacement. TM stem cells, mesenchymal stem cells (MSC) and induced pluripotent stem cells (iPSC)-derived TM cells promoted TM regeneration and in some cases also alleviated the IOP in models of OAG [15–18]. To realize the potential of such therapies it is critical to establish means of generating functional TM cells in vitro; newly discovered stem cells of the TM are enabling this potential. We and others recently established methodologies to isolate and propagate stem cells from the TM [19, 20]. We established that these progenitors display the defining characteristics of mesenchymal stem cells (MSC) [21], and termed them TM-derived MSC (TM-MSC). In our quest for cell replacement therapy for POAG, we are interested in establishing an expandable source of functional TM cells from their progenitors. To this end, we are establishing methods to direct the differentiation of TM-MSC into functional TM tissue. An impediment to this effort is the lack of cellular markers that are required to monitor differentiation of the cells and to characterize their purity.

Since no single definitive marker is available to identify TM cells, a combination of genes, highly expressed in the TM, is utilized to assign TM lineage (Table 1). Expression analysis found high expression of aquaporin 1 (AQP1) in primary human TM cells [22]. Gene expression profiling of TM cells followed by comparative analysis with Schlemm’s canal endothelium and subsequent reporter construct activity demonstrated the specificity of chitinase 3-like 1 (*CHI3L1)* to the TM in perfused anterior segments [23]. Similarly, matrix gla protein (*MGP)* was identified to be highly expressed in the TM [24–26]. Myocilin (*MYOC)* is also reported to be highly expressed in the TM with its expression alterable by glucocorticoids and topography among other factors [27]. In fact, this gene was first discovered and characterized in the TM as trabecular meshwork-inducible glucocorticoid response (*TIGR*) gene [28–30]. Matrix metallopeptidase 1 (*MMP1)* and ankyrin 3 (*ANK3*) are also among genes that have high expression in primary TM cells compared to cultured Schlemm’s canal endothelial cells and scleral fibroblasts as deduced by gene expression profiling [31]. Another marker, ELAM1 is expressed in glaucomatous TM cells [32]. These markers are commonly used collectively to distinguish the TM. In most instances, their specificity to the TM in the context of the anterior segment has not been demonstrated and it is likely that they are also highly expressed in the surrounding cell types. Moreover, complete molecular dissection of genes differentially expressed between the TM progenitors and mature TM cells has not been explored.

**Table 1:** Common TM markers identified by other groups

Gene	Method of identification	Reference
*CH3L1*	Gene expression profile analysis between human cultured TM cells and SC endothelial cells	Liton et al., 2005 [23]
*MGP*	Gene expression profiling of human TM cDNA library	Gonzalez et al., 2000 [24]; Tomarev et al., 2003 [25]; Gonzalez et al., 2004 [26]
*ANKG*	Gene expression profile analyses between human cultured TM cells, SC endothelial cells and scleral fibroblasts	Challa et al., 2003 [31]
*AQP1*	RT-PCR and immunofluorescence in human cultured TM cells	Stamer et al., 1995 [22]
*MMP1*	Gene expression profile analyses between human cultured TM cells, SC endothelial cells and scleral fibroblasts	Challa et al., 2003 [31]
*MYOC*	Identified in human TM cells as TIGR gene	Polansky et al., 1989 [28]
*ELAM1*	Expression detected in glaucomatous human TM cells by immunohistochemical screening	Wang et al., 2001 [32]

To identify suitable markers, genome-wide expression profiling of *ex vivo* TM tissue and cultured TM progenitors was performed to reveal genes differentially expressed between the mature and progenitor cells of the TM. To qualify as good differentiation markers, the genes have to be (1) differentially expressed between the TM progenitors and mature TM cells and (2) ideally specific to the TM in the anterior segment of the eye. From this study, we identified a panel of differentiation markers for the identification of mature TM cells as well as indicators of TM differentiation.

## Methods

### Sample collection

Research-grade corneoscleral tissues from deidentified human cadaver donors were procured from the Lions Eye Institute for Transplantation and Research (Tampa, FL, USA) and handled in accordance to the tenets of the Declaration of Helsinki. Informed written consent was attained from donors who were then registered on the local organ and tissue donor registry. For unregistered donors, written consent was acquired from the next of kin of the deceased donor. All donors were within 50–70 years of age and had no known record of chronic illnesses. Serological tests ensured that the samples were free of transmissible diseases. The corneoscleral specimens were harvested within 24 h of death, preserved in Optisol-GS at 4 °C and processed for the study within 13 days from preservation.

### Extraction of tissues

The corneoscleral specimen was washed several times in phosphate-buffered saline (PBS, Gibco; Thermo Fisher Scientific, Carlsbad, CA, USA) and stained with 0.1% trypan blue (Sigma, St. Louis, MO, USA) for 1 min. The dye stains the TM and scleral spur intensely with limited coloration of the corneal endothelium. After removal of excess stain with PBS rinses, the tissues were harvested by a technique adapted from Tripathi and Tripathi [33] in a moist chamber under a stereomicroscope taking sterile precautions. The corneal and scleral samples were extracted by making parallel cuts anterior to the Schwalbe’s line and posterior to the scleral spur respectively. The tissues were later minced into smaller pieces and homogenized in Trizol reagent (Ambion; Thermo Fisher Scientific) for RNA isolation. For TM extraction, the surrounding corneal endothelium and scleral spur were peeled off, and the TM was gently dissected from the underlying tissue with a crescent knife. The harvested TM was further processed for the isolation of TM progenitors or homogenized in Trizol reagent for gene expression studies by passing the sample several times through a blunt 20-gauge needle fitted to a nuclease-free syringe.

### Isolation and propagation of TM progenitors

TM-MSC culture was established from the harvested TM tissue as previously described [19]. Briefly, the TM tissue was digested with 2 mg/ml type I colleganese (Worthington Biochemical Corporation, Lakewood, NJ, USA) overnight at 37 °C in a humidified atmosphere of 5% CO_2_ in the cell culture incubator. The tissue was further dissociated in 0.05% trypsin-EDTA and triturated into a single cell suspension. The cells were then plated in low glucose DMEM containing L-glutamine and 100 mg/l sodium pyruvate, 10% fetal bovine serum (FBS), 10 mM non-essential amino acids, 100 units/ml Pencillin and 100 μg/ml Streptomycin. FBS and all other reagents were purchased from Gibco. Medium was changed every 2–3 days. The cells were passaged with 0.05% trypsin-EDTA when a confluence of 80–90% was reached and seeded at a ratio of 1:4. Characterization of the TM-derived cells as MSC was performed by flow cytometric analyses for MSC markers with accordance to the ISCT criteria [21]. Early passage cells at p3 – p4 were utilized for the study.

### RNA isolation

Total RNA was extracted from the Trizol/cell lysate followed by purification and DNase I treatment with the RNeasy miniprep kit (Qiagen, Venlo, Netherlands). Briefly, phase separation was performed by adding 200 μl chloroform per 1 ml of Trizol and after 3 min incubation, the mixture was centrifuged at 12 000 g for 15 min at 4 °C. The aqueous phase was then mixed with one volume of 70% ethanol and transferred to a RNeasy spin column with collection tube for the RNA to be purified and eluted according to the manufacturer’s protocol with an additional step of in-column DNase I treatment. RNA yield was determined on the NanoDrop ND-1000 spectrophotometer (Thermo Fisher Scientific). RNA quality was confirmed with the Agilent RNA 6000 pico kit on the Agilent 2100 Bioanalyzer (Agilent Technologies, Santa Clara, CA, USA).

### Microarray

Tissue samples obtained from three donors and TM-MSC isolated from three other donors were utilized for the study. RNA harvested from the samples was amplified with the TotalPrep RNA amplification kit according to manufacturer’s protocol (Ambion). The resultant purified biotin-labelled complementary RNA (cRNA) was quantified and 750 ng cRNA was applied to the HumanRef-8 v3.0 expression beadchip (Illumina, San Diego, CA, USA) following the direct hybridization instructions. Cy3 conjugated to streptavidin was used to detect the hybridized cRNA. The chip was scanned on the BeadArray Reader and imaged with the BeadScan software (both from Illumina).

The raw data were background-adjusted (as calculated from the negative control probes) and converted into expression profile by GenomeStudio (Illumina), and further analyzed with GeneSpring GX v14.5 (Agilent Technologies). The data were normalized by percentile shift to 75th percentile and threshold raw signal was set as 10. Unsupervised hierachical clustering was used to visualize gene expression similarity. The individual microarrays for the respective sample set were averaged and pairwise analysis was performed in comparison to TM, so relative intensity is the fold change relative to TM. Differentially expressed genes were analysed by one-way ANOVA, with correction for Benjamini & Hochberg false discovery rate (FDR) test. Data were filtered using fold change cut-off of 2.0 and *P*_adj_ ≤ 0.05 (*q*-value; defined as statistical significance). Entity lists generated from the analyses are available under Additional file 1. Our data have been deposited in NCBI's Gene Expression Omnibus (GEO) repository and accessible through GEO series accession number GSE87526.

### Quantitative real-time polymerase chain reaction

cDNA was generated from RNA extracted from tissues derived from three more donors and three other independent TM-MSC lines using the high-capacity cDNA reverse transcription kit (Applied Biosystems, Foster City, CA, USA) according to prescribed protocol. Quantitative real-time polymerase chain reaction (qPCR) was performed by direct dye binding utilizing SYBR Green (Applied Biosystems). Primers were designed by Primer3 [34, 35] or PrimerBank [36] with an annealing temperature of 55 °C where possible (Additional file 2: Table S1). cDNA was analysed in duplicates on the 7900HT Fast Real-Time PCR system and analyzed using SDS 2.4 software (both from Applied Biosystems). Amplification of *ACTB* was performed for each cDNA for normalization of RNA content. Relative mRNA abundance was calculated by the ∆∆CT method of comparative quantification. All values were presented as mean ± standard error of the mean (mean ± SEM) from three biological replicates. Significance was assessed using Student’s *t-test* and significant differences were considered as those with *P <* 0.05.

### Immunofluorescence

The corneoscleral specimen was washed several times in PBS to remove detached cells. The excess conjunctiva was removed with an angular iris dissecting scissors and the specimen was cut in half. The cornea and sclera were trimmed and the sample comprising the corneoscleral limbus was embedded in the optimal cutting temperature (OCT) compound (Tissue-Tek; Sakura Finetek USA, Torrance, CA, USA) in a cryomold and frozen at −80 °C. The frozen tissue sample was cut into 8 μm sections at the cryostat (Leica, Wetzlar, Germany), mounted onto microscope slides (Thermo Fisher Scientific), air-dried for 15 min and stored at −80 °C. The cryosections were thawed at room temperature and rehydrated with water before proceeding to fixation and permeabilization.

The cryosections were washed in PBS and fixed in 4% paraformaldehyde (PFA) (Sigma) pH 7.4 for 10 min at room temperature. The tissue sections were then washed twice in PBS for 5 min and permeabilized in 0.25% Triton X-100 (Sigma) for 10 min, followed by three washes. To block non-specific binding of antibodies, the samples were incubated with 10% heat-inactivated goat serum and 0.3 M Glycine (Sigma) in PBST for 30 min. The sections were then incubated in primary antibody, diluted at optimized concentration in PBST containing 1% serum, overnight at 4 °C and washed thrice. Alexa Fluor 594-conjugated secondary antibody in 1% serum was added and incubated for 1 h in the dark at room temperature and washed three times. Nuclei were stained with 1 μg/ml 4′,6-diamidino-2-phenylindole (DAPI) (Invitrogen, Carlsbad, CA, USA) for 1 min followed by several washes. Finally, coverslip was mounted onto the glass slide with fluorescence mounting medium (Dako, Denmark) and affixed with nail polish. Images were acquired under the camera mode of the fluorescence microscope (Zeiss, Germany). Images were archived under the Zeiss AxioVision 4.8 image analysis software and processed in Adobe Photoshop (Adobe Systems, San Hose, CA, USA). Independent cohort of cornealscleral specimens were used for the study. Details of the antibodies used for the study are listed in Additional file 2: Table S2.

## Results

### Expression patterns of known TM markers

**Fig. 1:**
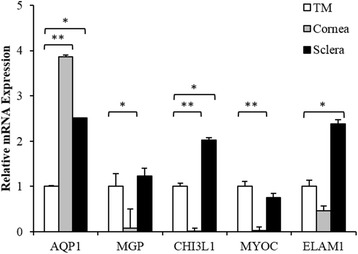
Expression profiling of previously reported TM markers in the TM tissue, cornea and sclera. qPCR analysis of several TM markers found them to be have higher expression in the cornea and sclera relative to TM (*AQP*1) or highly expressed in both the TM and sclera (*MGP*, *CHI3L1* and *ELAM1*). *MYOC* showed highest expression in the TM among the three tissues. Error bars show SEM of triplicate analyses. ** *P* < 0.01; * *P* < 0.05 (Student’s *t*-test)

Expression studies by other groups have identified several genes highly expressed in the TM, which have been used as markers of the TM. To assess the specificity of their expression in the TM relative to the peripheral tissues of cornea and sclera, we performed qPCR analysis with tissues derived from three donors. Surprisingly, expression of *AQP1* was much higher in the adjacent tissues relative to TM (Fig. 1). The remaining markers, *MGP*, *CHI3L1* and *ELAM1* had greater expression in the TM relative to cornea, but generally showed higher expression in the sclera. Only *MYOC* was highly expressed in the TM compared to the peripheral tissues but not significant statistically compared to sclera (*P* ≥ 0.05). Given that previously identified TM markers are lacking in specificity, additional markers are needed.

### Gene expression profiling of TM-MSC and TM

In this study we sought to identify robust differentiation markers of the TM. To this end, we searched for genes that display high differential expression between the TM-MSC and mature TM cells. E*x vivo* TM, mostly comprised of differentiated TM cells, was utilized as a source of mature TM cells. To identify genes with differential expression, gene expression profiling by microarray technology was performed on TM tissue and TM-MSC derived from three independent donors of the same age group (50–70 years). Signal intensities were normalized by the method of percentile shift (75th) and differential genes that passed the fold change cut-off of 2.0 with statistical significance, as determined by one-way ANOVA followed by Benjamini-Hochberg multiple testing corrected *P* ≤ 0.05 were identified.

**Fig. 2:**
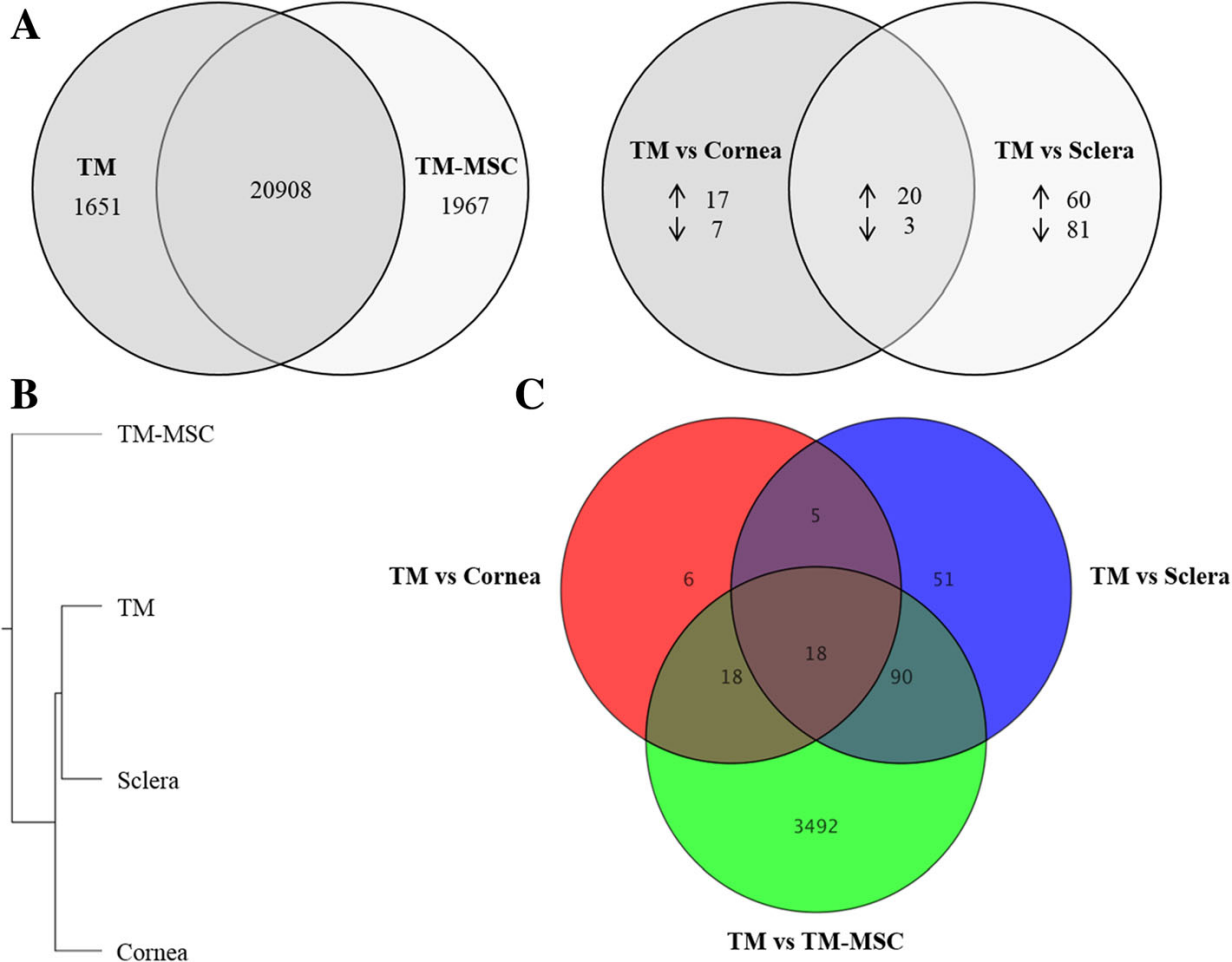
Genome-wide expression profiling of TM, TM-MSC, cornea and sclera. **a** Venn diagram indicates number of genes elevated in the TM and TM-MSC with respect to each other as determined by their comparative analysis (*left*). Venn diagram of genes differentially expressed between TM vs cornea and TM vs sclera (*right*). 22 genes were differentially expressed in the TM relative to cornea and sclera. (Statistical threshold of fold change ≥ 2.0 and *P*_adj_ ≤ 0.05 were applied for both). **b** Hierarchical clustering of the sample sets based on whole transcriptome profile showed that the tissues shared some extent of expression similarity, with sclera sharing closer similarity to the TM. The TM-MSC profile was divergent from the tissues. **c** Venn diagram designed by merging the gene lists (TM vs TM-MSC/cornea/sclera) found 18 genes to be differentially expressed in the TM compared to the other sample sets under the statistical threshold selected

The resultant entity list had a considerable number of genes differentially expressed between the two sample sets, with 1651 and 1967 probes corresponding to those genes elevated in the TM and TM-MSC, respectively (Fig. 2a). *MYOC* and angiopoietin-like 7 (*ANGPTL7)* were most elevated in the TM, being over 1000-fold higher in the TM compared to TM-MSC. The top 20 genes elevated in the TM are listed in Table 2. These genes code for proteins expressed in the different cell compartments, such as intracellular proteins (*PCP4*, *KRT13*, *KRT5*, *MYH11*, *HBB*, *FAM107A*), cell surface proteins (*CDH23*, *HLA-DRα*, *VCAM1*, *CXADR*, *TACSTD2*), and secreted proteins (*MYOC*, *ANGPTL7*, *CCL3L3*, *SERPINA3*, *APOD*, *FCGBP*, *PTGDS*, *C2ORF40*). *H19*, a long non-coding RNA was also elevated.

**Table 2:** Top 20 genes highly expressed in the TM compared to TM-MSC

Symbol	Definition	|Fold Change| (TM/TM-MSC)	*P*_adj_
*MYOC*	Myocilin, trabecular meshwork inducible glucocorticoid response	1374	6.97E-04
*ANGPTL7*	Angiopoietin-like 7	1189	1.48E-03
*PCP4*	Purkinje cell protein 4	656	2.33E-03
*KRT13*	Keratin 13	459	1.08E-03
*SERPINA3*	Serpin peptidase inhibitor, clade A (alpha-1 antiproteinase, antitrypsin), member 3	447	3.75E-03
*MYH11*	Myosin, heavy chain 11, smooth muscle	429	1.00E-02
*CDH23*	Cadherin-like 23	420	6.97E-04
*HLA-DRA*	Major histocompatibility complex, class II, DR alpha	354	9.27E-04
*PTGDS*	Prostaglandin D2 synthase 21 kDa (Brain)	300	2.05E-02
*FAM107A*	Family with sequence similarity 107, member A	275	6.97E-04
*HBB*	Hemoglobin, beta	264	4.13E-03
*C2orf40*	Chromosome 2 open reading frame 40	239	8.31E-04
*FCGBP*	Fc fragment of IgG binding protein	231	9.27E-04
*APOD*	Apolipoprotein D	230	2.40E-02
*CXADR*	Coxsackie virus and adenovirus receptor	218	7.76E-04
*TACSTD2*	Tumor-associated calcium signal transducer 2	215	9.45E-04
*H19*	H19, imprinted maternally expressed transcript (non-protein coding)	210	1.38E-03
*KRT5*	Keratin 5	209	7.76E-04
*CCL3L3*	Chemokine (C-C motif) ligand 3-like 3	209	1.27E-03
*VCAM1*	Vascular cell adhesion molecule 1	199	2.49E-03

Interestingly, *HLA-DRα* which we showed previously is not expressed in TM-MSC was highly expressed in the mature cells [19]. *THY-1* (*CD90*), a MSC marker present in TM-MSC, was repressed in the TM. TM-MSC expressed alpha-kinase 2 (*ALPK2*) and lysyl oxidase (*LOX*) at the greatest level compared to the TM, at 137- and 71-fold, respectively. Other genes highly repressed in the TM are listed in Table 3.

**Table 3:** Top 20 genes highly expressed in the TM-MSC relative to TM

Symbol	Definition	|Fold Change| (TM-MSC/TM)	*P*_adj_
*ALPK2*	Alpha-kinase 2	137	3.03E-03
*LOX*	Lysyl oxidase	71	7.83E-03
*FOXD1*	Forkhead box D1	67	5.36E-03
*STS1*	Cbl-interacting protein Sts-1	61	2.52E-03
*STC2*	Stanniocalcin 2	59	5.35E-03
*CD151*	CD151 molecule (Raph blood group)	57	3.55E-03
*KIAA0101*	KIAA0101	49	7.10E-03
*KCNK2*	Potassium channel, subfamily K, member 2	49	7.86E-03
*RPLP0*	Ribosomal protein, large, P0	47	3.82E-03
*MELK*	Maternal embryonic leucine zipper kinase	44	5.18E-03
*MARCH4*	Membrane-associated ring finger (C3HC4) 4, E3 ubiquitin protein ligase	43	4.56E-03
*TXNRD1*	Thioredoxin reductase 1	42	1.59E-02
*TMEM132D*	Transmembrane protein 132D	41	1.20E-03
*THY1*	Thy-1 cell surface antigen	40	1.56E-03
*TNFRSF11B*	Tumor necrosis factor receptor superfamily, member 11b	39	1.22E-02
*FN1*	Fibronectin 1	37	1.05E-03
*WDR1*	WD repeat domain 1	36	1.54E-03
*KDELR3*	KDEL (Lys-Asp-Glu-Leu) endoplasmic reticulum protein retention receptor 3	36	6.97E-04
*TSPO*	Translocator protein (18 kDa)	36	2.49E-03
*RAB3B*	RAB3B, member RAS oncogene family	34	8.98E-03

We analysed our data to assess whether previously reported TM markers were differentially expressed between the mature TM cells and TM-MSC. *MYOC* and *MGP* were robustly elevated in the TM at 1374- and 67-fold, respectively. Expression of *AQP1* was about 4-fold higher in the TM. The remaining markers, *CHI3L1, MMP1* and *ELAM1,* were not found in the list of differentially expressed genes. Further investigation was done to determine whether the differentially expressed markers are specific to the TM region of the eye.

### Gene expression profiling of cornea and sclera

To find genes uniquely expressed in the TM in the context of the anterior segment of the eye, corneal and scleral tissues, harvested from three donor specimens, were included in the microarray study and pairwise analysis relative to the TM was performed as described earlier. The resulting gene expression data were processed into two entity lists: TM vs cornea and TM vs sclera (fold difference ≥ 2.0 and *P*_adj_ ≤ 0.05). A limited number of genes were found to have differential expression: 47 genes in the TM vs cornea and 164 in the TM vs sclera data sets. Both gene lists were merged to determine genes that are differentially expressed in the cornea and sclera in common with respect to the TM (i.e. TM vs cornea/sclera) (Fig. 2a). Twenty genes had significantly higher expression in the TM and three were lower in comparison to the cornea and sclera (Table 4).

**Table 4:** Genes differentially expressed in the cornea and sclera with respect to the TM

	TM/Cornea			TM/Sclera		
Gene	|Fold Change|	Regulation	*P*_adj_	|Fold Change|	Regulation	*P*_adj_
*F5*	29	up	3.40E-02	48	up	8.49E-03
*NEB*	27	up	3.34E-02	29	up	1.80E-02
*CALB2*	27	up	3.34E-02	34	up	3.12E-02
*SORCS1*	20	up	3.34E-02	7	up	1.80E-02
*BDNF*	18	up	3.40E-02	22	up	8.49E-03
*KCNAB1*	16	up	4.04E-02	8	up	1.73E-02
*TMEM178*	14	up	3.62E-02	10	up	3.73E-02
*SPP1*	12	up	3.40E-02	8	up	4.14E-02
*FGF9*	12	up	4.68E-02	14	up	3.34E-02
*HEY1*	10	up	4.95E-02	6	up	1.80E-02
*CD1D*	9	up	4.95E-02	10	up	2.42E-02
*CDH23*	9	up	3.62E-02	5	up	3.82E-02
*CDH2*	8	up	4.95E-02	14	up	4.10E-02
*MGC33846*	7	up	3.40E-02	6	up	3.63E-02
*ZBTB46*	6	up	3.40E-02	3	up	3.73E-02
*LOXL3*	6	up	3.40E-02	4	up	3.73E-02
*SLC16A10*	5	up	3.40E-02	9	up	1.96E-02
*SYTL4*	4	up	1.83E-02	3	up	8.49E-03
*FAIM2*	4	up	3.34E-02	6	up	3.79E-02
*RAPGEF1*	2	up	4.95E-02	4	up	1.96E-02
*BCL11B*	7	down	4.95E-02	5	down	3.82E-02
*SLC7A2*	3	down	4.31E-02	3	down	3.34E-02
*ITPA*	2	down	4.95E-02	2	down	3.73E-02

Surprisingly, none of the previously identified TM markers were present in the merged TM vs cornea/sclera gene list, indicating that they were not specific to the TM. Hence, our approach was modified to discover new markers from this gene set that are also differentially expressed between the mature TM cells and TM-MSC. This could identify genes that are both ‘TM-specific’ in the context of the anterior segment and robust enough to track TM differentiation.

### Identification of trabecular meshwork differentiation markers

Unsupervised hierarchical clustering showed that the tissues clustered together, apart from TM-MSC, with the TM having greater extent of expression similarity to sclera than cornea (Fig. 2b). This confirms the divergent state of the differentiated cells in the tissues from the progenitor state of TM-MSC. Overlap of the three gene lists (TM vs TM-MSC, TM vs cornea and TM vs sclera) was performed to identify TM unique genes. From the comparative analyses, 18 genes were found to be differentially expressed in the TM tissue compared to TM-MSC, cornea and sclera (Fig. 2c). Specifically, 16 genes (namely *CDH23*, *SLC16A10*, *SPP1*, *F5*, *KCNAB1*, *MGC33846*, *FGF9*, *TMEM178*, *HEY1*, *NEB*, *SORCS1*, *CD1D*, *ZBTB46*, *SYTL4*, *FAIM2* and *BDNF*) were elevated and two were repressed (*ITPA* and *SLC7A2*) in the TM tissue relative to the other tissues. Similar trend was observed in the TM vs TM-MSC gene set, except BDNF which was repressed in the TM. Details of the genes, fold change and regulation are presented in Table 5.

Six genes elevated in the TM were selected for further analysis based on largest divergence in expression from the cornea and sclera, and availability of antibodies. Cadherin-related 23 (*CDH23*), secreted phosphoprotein 1 (*SPP1*), coagulation factor 5 (*F5*), potassium voltage-gated channel, shaker-related subfamily, beta member 1 (*KCNAB1*), fibroblast growth factor 9 (*FGF9*), and hairy/enhancer-of-split related with YRPW motif 1 (*HEY1*) were chosen as TM differentiation markers (Table 5). *CDH23* had the highest differential expression (420-fold) in the TM relative to TM-MSC. Its expression in the TM in comparison to the cornea and sclera were 9- and 5-fold, respectively. The other selected genes were elevated in the TM by fold differences of 35–100 compared to TM-MSC, and 5–50 relative to the cornea/sclera. As stated earlier, brain-derived neurotropic factor (*BDNF*) was the only gene repressed in the TM tissue relative to TM-MSC in the merged gene list and thus selected as a TM-MSC marker (i.e. negative differentiation marker). While *BDNF* was repressed by 13-fold in the TM in comparison to TM-MSC, its expression was higher in the TM tissue relative to the adjacent tissues.

**Table 5:** List of genes differentially expressed in the TM compared to TM-MSC, cornea and sclera. Genes highlighted in bold were shortlisted for further characterization

	TM-MSC		Cornea		Sclera	
Gene	|Fold Change|	Regulation	|Fold Change|	Regulation	|Fold Change|	Regulation
***CDH23***	420	up	9	up	5	up
*SLC16A10*	87	up	5	up	9	up
***SPP1***	85	up	12	up	8	up
***F5***	66	up	29	up	48	up
***KCNAB1***	56	up	16	up	8	up
*MGC33846*	42	up	7	up	6	up
***FGF9***	40	up	12	up	14	up
*TMEM178*	40	up	14	up	10	up
***HEY1***	36	up	10	up	6	up
*NEB*	35	up	27	up	29	up
*SORCS1*	26	up	20	up	7	up
*CD1D*	10	up	9	up	10	up
*ZBTB46*	8	up	6	up	3	up
*SYTL4*	8	up	4	up	3	up
*FAIM2*	5	up	4	up	6	up
***BDNF***	13	down	18	up	22	up
*ITPA*	5	down	2	down	2	down
*SLC7A2*	3	down	3	down	3	down

Verification by qPCR was performed to confirm the microarray results. Expression of the shortlisted differentiation markers was assessed in tissues obtained from three new donors and three other TM-MSC lines. Deriving the samples from new biological replicates increases the reliability of the study by eliminating donor or batch specific variations.

**Fig. 3:**
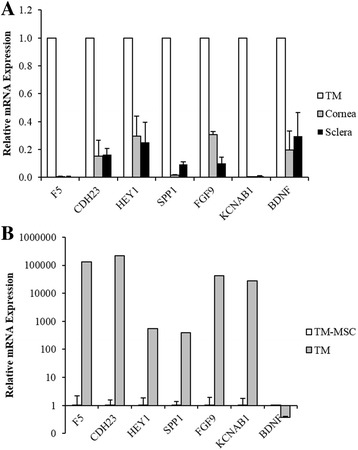
Expression analysis of shortlisted marker genes by qPCR. **a** The genes were all significantly repressed in the cornea and sclera relative to the TM. **b** The positive markers were highly expressed in the TM, while *BDNF* (negative marker) was low, in comparison to TM-MSC. Error bars show SEM of triplicate analyses. All differences were statistically significant at *P* < 0.05 (Student’s *t*-test)

qPCR result confirmed the repression of the differentiation markers in the cornea and sclera compared to the TM (Fig. 3a). *F5* had the lowest expression in the two peripheral tissues followed by *KCNAB1* with fold difference close to 0.01 relative to the TM (*P* < 0.0001). Remaining markers were also significantly repressed with fold changes between 0.1 and 0.3 (*P* < 0.05). This confirms higher expression of the selected markers in the TM relative to the cornea and sclera.

In comparison to TM-MSC, the positive differentiation markers were all expressed significantly higher in the TM, while *BDNF* was repressed (*P* < 0.05) (Fig. 3b). The positive markers were more than 100-fold higher, with *F5* and *CDH23* having strongest expression in the TM relative to TM-MSC. *BDNF* was substantially lower at 0.3-fold in the TM. The qPCR results from independent samples confirmed the differential expression of the identified markers in the two cell types. Thus, from the mRNA expression analysis, the markers are: 1) specific to the TM relative to the cornea and sclera; and 2) robustly differential in expression between the TM and TM-MSC.

### Immunofluorescence of selected markers in the TM

**Fig. 4:**
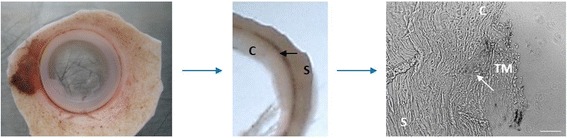
Preparation of corneoscleral tissue sections. Anterior segment of donor eye, trimmed off excess cornea and sclera, was embedded in OCT compound and cryosectioned transversely. Representative brightfield image of a section is presented. Components of the anterior segment are annotated as follows: cornea (C), corneoscleral junction (*black arrow*), sclera (S), TM and schlemm’s canal (*white arrow*). Scale bar: 100 μm

**Fig. 5:**
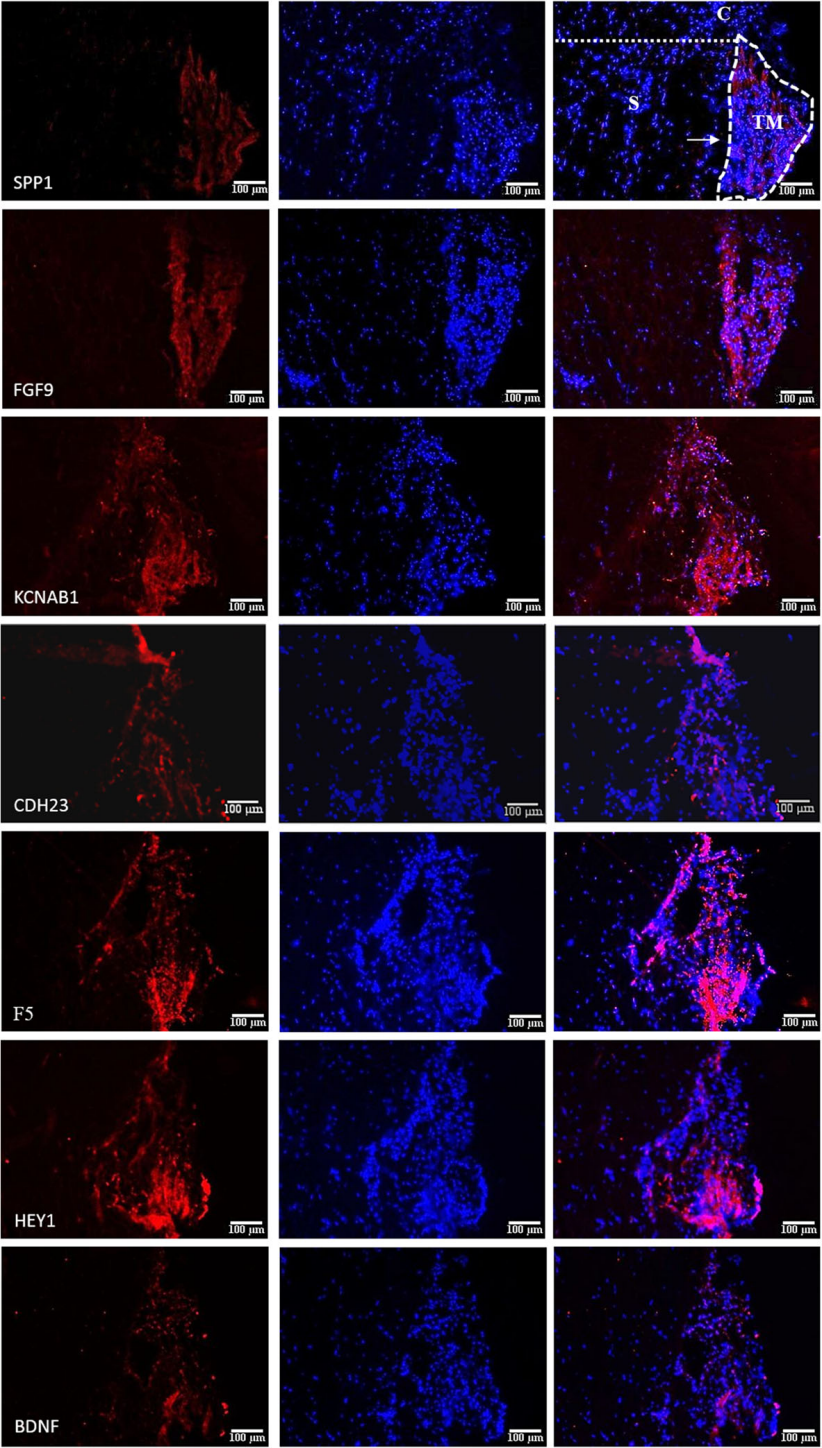
Immunofluorescence of TM differentiation markers on corneoscleral sections. The positive markers were intensely stained in the TM compared to the rest of the anterior segment. The negative marker, BDNF, was also slightly more intense in the TM relative to peripheral tissues. Components of the anterior segment are annotated as follows: cornea (C), sclera (S), TM and Schlemm’s canal (*arrow*). Scale bar: 100 μm

To ensure the markers are highly expressed in the TM compared to the rest of the anterior segment, immunofluorescence was performed on corneoscleral tissue. Corneoscleral tissue sections were prepared from donor eyes by trimming off the excess cornea and sclera, embedded in OCT compound and cryosectioned transversely (Fig. 4). Frozen sections were fixed and permeablized, followed by immunostaining with commercially available antibodies to assess the expression of markers in the TM and surrounding tissues. Two markers, SPP1 and FGF9, stained brightly in the TM and were undetectable in the surrounding tissues (Fig. 5). Other positive markers of TM differentiation, KCNAB1, CDH23, F5 and HEY1 were clearly detected in the TM, though a weak signal was also detected in peripheral tissues, particularly in the sclera, and in some cases in the Schlemm’s canal. BDNF which was elevated in the TM relative to the cornea and sclera at the transcript level, was of stronger intensity in the TM compared to the surrounding tissues, but not as pronounced as the positive markers. As a TM-MSC marker, BDNF was expected to be expressed most intensely in the ‘insert portion’ of the TM, a region under the Schwalbe’s line of the cornea where TM progenitors are believed to reside. However, this was not apparent probably due to the small number of stem cells in this region. Corresponding staining in the TM-MSC showed negligible signal for all the TM markers, while BDNF expression was detected (Additional file 3: Figure S1).

## Discussion

The pathophysiological mechanisms causing POAG are not completely understood. However, the association of TM dysfunction with POAG, as characterized by structural changes in the TM, has been well established. Since, the structural and functional integrity of the TM is indispensable in maintaining normal IOP and healthy vision, cellular therapy is being explored to replace the lost or dysfunctional cells with healthy new ones [15, 16]. The capacity of progenitors of the TM to integrate into the TM tissue and differentiate has already been demonstrated [15].

Expression of a combination of genes highly expressed in the TM is generally accepted to identify the TM cells, however most currently known markers are also expressed in other ocular cells. In our attempt to find markers better suited as TM differentiation markers to track differentiation from their progenitors, we generated a list of genes differentially expressed in the TM-MSC and *ex vivo* TM tissue by comprehensive transcriptome profiling. To our knowledge, this is the first report dechipering molecular level differences between the mature TM cells and a pure population of its progenitors.

There are scattered reports on certain genes differentially expressed in the filtering compartment of the TM and its non-filtering stem cell niche. Through immunocharacterization in vivo, it was reported that ANK3 and HMFG1 are highly expressed in the niche, while CHI3L1 is low compared to the TM tissue [31]. Contrary to this report, we found that *ANK3* is upregulated in the TM compared to TM-MSC. In addition, *HMFG1* and *CHI3LI* were not differentially expressed. Several other genes highly expressed in the TM (*MYOC*, *AQP1*, *MGP* and *MMP1*) were higher in the TM relative to TM-MSC. However, they have similar expression in the cornea and sclera, rendering them non-specific to the TM.

*MYOC* and *ANGPTL7* were the most differentially expressed genes in the TM relative to TM-MSC. It had been shown previously by serial analysis of gene expression (SAGE) profiling that *MYOC* and *ANGPTL7* are among the 40 most highly expressed genes in the TM [37]. Besides the TM, MYOC expression was detected in the cornea, sclera, choroid, ciliary body, iris, lamina cribosa, retina and optic nerve as well as in the acellular vitreous and aqueous humour [38–41]. ANGPTL7 is highly expressed in the cornea and lens [42]. Its expression in other ocular tissues is not well understood. To elucidate markers that are robust and specific to the TM, genes enriched in the TM tissue in relation to cornea, sclera and TM-MSC were identified through comprehensive transcriptome profiling and selected candidates were further evaluated. Relative mRNA levels, as measured by qPCR, confirmed highest expression of the transcripts in the TM. Immunocharacterization on corneoscleral sections also demonstrated high marker expression in the TM. Some markers displayed specificity to the TM while the rest had greater expression in the TM in the context of the anterior segment of the eye. In addition, BDNF which was repressed in the TM in relation to TM-MSC was identified as a negative indicator of TM differention.

The newly identified markers of TM differentiation (TM markers) include secreted proteins F5, SPP1 and FGF9, membrane proteins CDH23 and KCNAB1, and transcription factor HEY1. F5, a key cofactor for prothrombinase activity, is part of the coagulation process. The gene has been mapped to the glaucoma locus, GLC1A where *MYOC* also resides and is known to be downregulated in cultured human TM cells [14]. Similarly SPP1, a phosphoprotein involved in matrix mineralization, is also downregulated in human TM cells in vitro and highly expressed in the TM tissue [24, 25, 43]. Primary TM cells in these studies will likely contain TM progenitors and repression of both proteins compared to the TM tissue support our data. SPP1 is also a cytokine that promotes interferon gamma (IFNG) and interleukin 12 (IL12) production, hence functioning in immune response [44, 45]. Other activities associated with SPP1 are migration, ECM remodelling, cell adhesion and survival [45]. Interestingly, it is an inhibitor of calcification like MGP, another well established marker of the TM [46]. FGF9 is involved in biological processes important for development, such as tissue repair, proliferation and differentiation of several cell types including induction of neural crest from which the TM was derived [47]. Receptors for FGF signalling have been detected in cultured human TM cells and their activation stimulates proliferation and extracellular acidification [48]. The association of these genes with TM function and maintenance is unknown.

CDH23 is a member of the cadherin superfamily that encodes calcium-dependent cell-cell glycoprotein. It plays an essential role in auditory function as mutations in the gene leads to deafness in humans and mice [49, 50]. As an integral part of stereocilia and hair bundle of cochlear hair cells [51, 52], it has a putative role in mechanotransduction. It is tempting to speculate that high expression of CDH23 in the TM may offer a similar mechanosensitivity to the tissue, allowing IOP homeostasis. It may also be involved in the structural integrity of the TM. KCNAB1 heterotetramerizes with alpha subunits and modulates the activity of the K_v_ channels [53–55]. It is involved in a range of functions including but not limited to regulating neurotransmitter release, heart rate, insulin secretion, smooth muscle contraction and cell volume. Transcriptional repressor, HEY1 which is activated by Notch signalling [56, 57], is important in development [58]. It is essential for cardiovascular development [59, 60], and implicated in neurogenesis [61], somitogenesis and osteogenesis [62]. The roles KCNAB1 and HEY1 play in the TM mechanism are yet to be known. BDNF which was recognized as a TM-MSC marker in this study is a neurotrophin involved in the regulation of survival, differentiation and stress response. It is known to be expressed by both cultured human TM cells and *ex vivo* TM tissue, with the former expressing it at a higher level [63]. *BDNF* polymorphism has also been implicated with POAG progression [64], although its exact pathophysiological mechanism is unclear.

The screening of markers was performed on samples attained from new sets of donors for the various expression studies, thereby minimizing batch-specific and donor-specific biases. Moreover, e*x vivo* TM used in the study instead of cultured primary TM cells offers a more accurate molecular dissection of the mature TM cells. Marker gene expression was investigated in a commercially available TM cell line by qPCR analysis; four of the markers (*F5*, *HEY1*, *FGF9* and *KCNAB1*) were found to be significantly elevated, while *BDNF* was repressed relative to TM-MSC (Additional file 4: Figure S2). We are attempting to establish conditions that induce TM differentiation from TM-MSC in vitro. From our preliminary results, we observed partial induction of *F5*, *CDH23*, *SPP1*, *HEY1, KCNAB1* and *FGF9,* indicating that these markers may be useful to monitor the process of differentiation towards TM lineage (unpublished data). Thus, this panel of markers together with previously established TM markers could allow rigorous assessment of differentiation in transplantation studies targeting the TM as well as in the generation of mature TM cells in vitro for molecular applications.

## Conclusions

Our study has identified a set of markers of TM differentiation. It is better to use the markers collectively rather than a single identifier to ensure robust differentiation. We have confirmed by independent means of expression analysis that these markers are: (1) differentially expressed between the TM and TM-MSC and 2) specific to the TM relative to other regions in the anterior segment. Upon successful differentiation of TM-MSC into TM lineage, the positive markers (TM markers) are expected to be upregulated, whereas BDNF being a negative indicator (TM-MSC marker) ideally should be downregulated.

## Additional files

Additional file 1: Data on the differential gene lists TM vs TM-MSC, TM vs cornea and TM vs sclera. (XLSX 721 kb)

Additional file 2: Table S1. List of primer sequences used in the study. **Table S2.** Details of antibodies utilized for immunofluorescence. (DOCX 13 kb)

Additional file 3: Figure S1. Immunofluorescence of TM markers on TM-MSC. (TIF 4091 kb)

Additional file 4: Figure S2. qPCR analysis of identified marker genes in TM cells (TMC) relative to TM-MSC. *** *P* < 0.001; ** *P* < 0.01; * *P* < 0.05 (Student’s *t*-test). (TIF 678 kb)
