# Supplementary material for: Transcriptome analysis for the identification of cellular markers related to trabecular meshwork differentiation
Source: BMC Genomics. 2017 May 17;18:383. doi: 10.1186/s12864-017-3758-7 (PMC5436446; doi:10.1186/s12864-017-3758-7)
Supplement: Supplementary file 2 — List of primer sequences used in the study. Table S2. Details of antibodies utilized for immunofluorescence. (DOCX 13 kb) [file 12864_2017_3758_MOESM2_ESM.docx]

**Table S1.** List of primer sequences used in the study.

| **Gene** | **Forward (5’ -> 3’)** | **Reverse (5’ -> 3’)** |
| --- | --- | --- |
| *AQP1* | CTGCACAGGCTTGCTGTATG | TGTTCCTTGGGCTGCAACTA |
| *MGP* | GCCGCCTTAGCGGTAGTAAC | TCTCTGCTGAGGGGATATGA |
| *CHI3L1* | CCTTGACCGCTTCCTCTGTA | GTGTTGAGCATGCCGTAGAG |
| *MYOC* | AAGCCCACCTACCCCTACAC | TCCAGTGGCCTAGGCAGTAT |
| *ELAM1* | ACACCTCCACGGAAGCTATG | AATTGCAACCAGGTGTGTGTA |
| *F5* | GACGTTTGACAAGCAAATCGTG | CATTAGGGATGATGACTGGCTC |
| *CDH23* | CGCCCACATTTCACAATCAGC | CGTCCCCACTGGTGTATTCT |
| *HEY1* | ATCTGCTAAGCTAGAAAAAGCCG | CGTCAAAGTAACCTTTCCCTCCT |
| *SPP1* | GTTTCGCAGACCTGACATCCA | GCTTTCCATGTGTGAGGTGAT |
| *FGF9* | CAGGCGGAGGCAGCTATAC | CCTGGTTCCCTGGATAGTACC |
| *KCNAB1* | ATACTGCCGAAGTCTATGCTGC | GAGGGAGCCCTTCAATCCTT |
| *BDNF* | GGTTTCATAAAGTTCCACCAG | GGATGTTTGCTTCTTTCATGG |

**Table S2.** Details of antibodies utilized for immunofluorescence.

| **Antibody** | **Protein** | **Company (Catalogue No.)** |
| --- | --- | --- |
| Primary | F5 | Santa Cruz Biotechnology (sc-66041) |
| Primary | CDH23 | Abcam (ab131135) |
| Primary | HEY1 | Abcam (ab22614) |
| Primary | SPP1 | Abcam (ab63856) |
| Primary | FGF9 | Abcam (ab71395) |
| Primary | KCNAB1 | Abcam (ab156700) |
| Secondary | Goat anti-Mouse Alexa Fluor 594 | Thermo Fisher Scientific (A-11005) |
| Secondary | Goat anti-Rabbit Alexa Fluor 594 | Thermo Fisher Scientific (A-11037) |
