# Supplementary figures and images for: Transcriptome analysis for the identification of cellular markers related to trabecular meshwork differentiation
Source: BMC Genomics. 2017 May 17;18:383. doi: 10.1186/s12864-017-3758-7 (PMC5436446; doi:10.1186/s12864-017-3758-7)

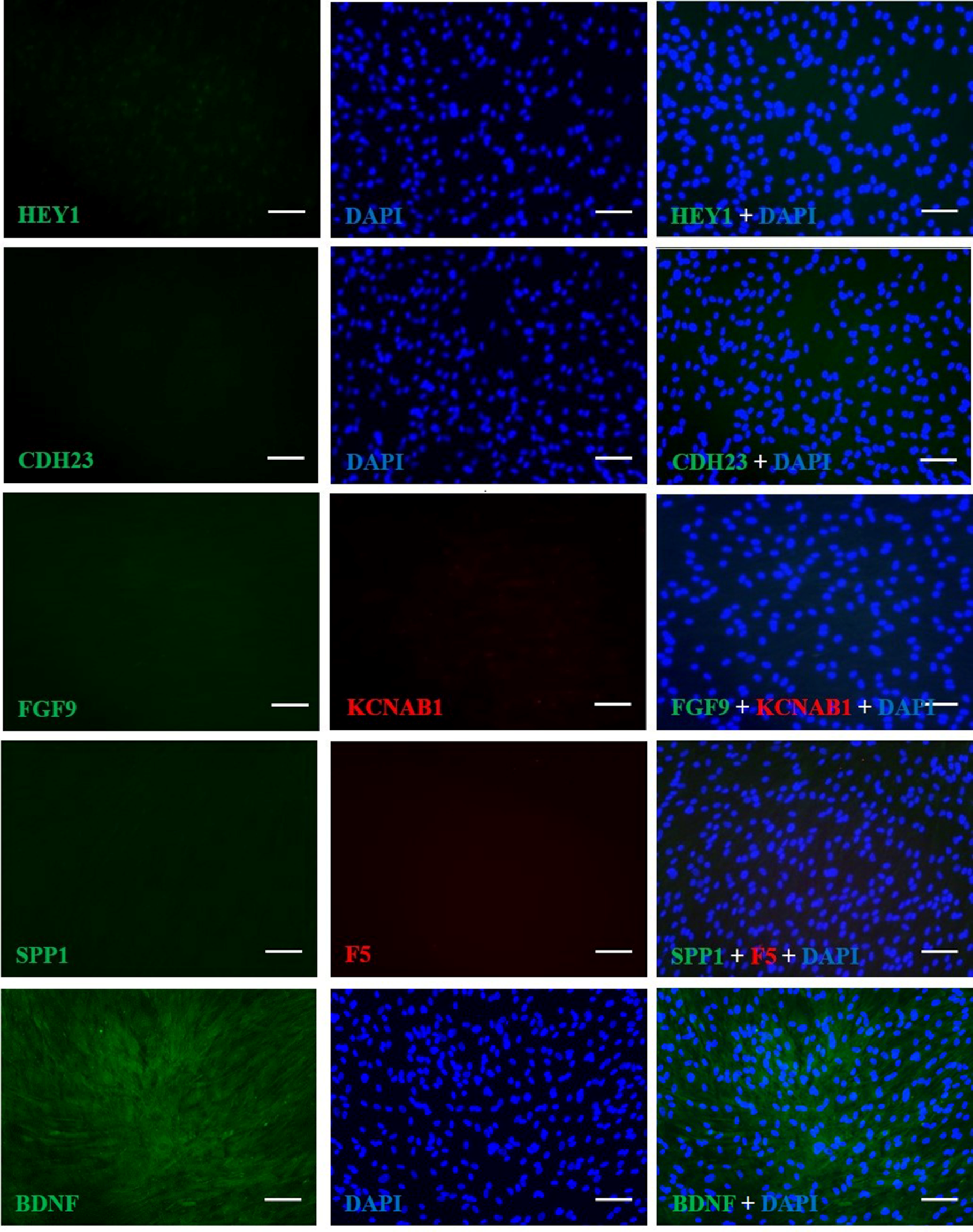

Supplement: Supplementary file 3 — Immunofluorescence of TM markers on TM-MSC. (TIF 4091 kb) [file 12864_2017_3758_MOESM3_ESM.tif]

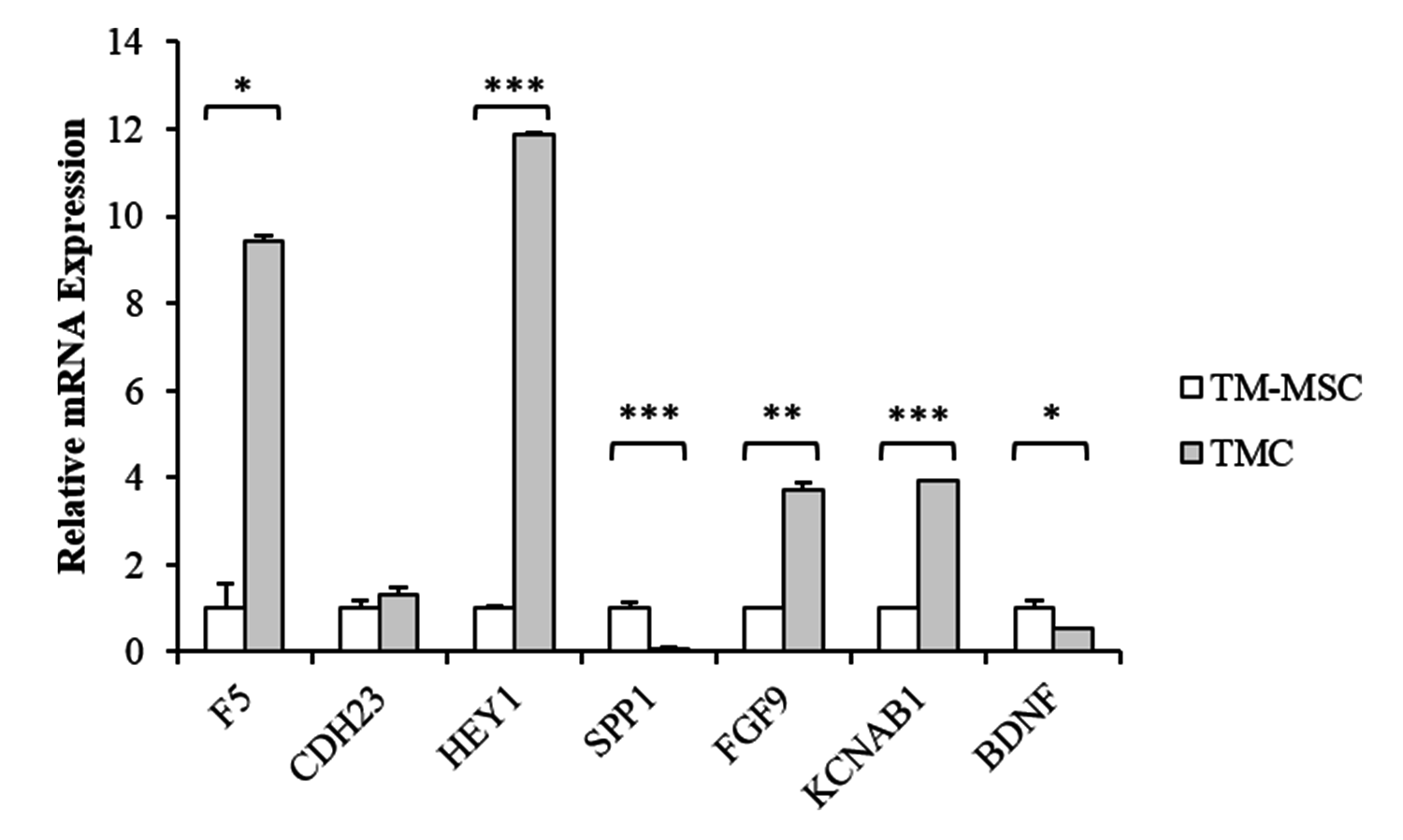

Supplement: Supplementary file 4 — qPCR analysis of identified marker genes in TM cells (TMC) relative to TM-MSC. *** P < 0.001; ** P < 0.01; * P < 0.05 (Student’s t-test). (TIF 678 kb) [file 12864_2017_3758_MOESM4_ESM.tif]
